# Early musical training benefits to non-musical cognitive ability associated with the Gestalt principles

**DOI:** 10.3389/fpsyg.2023.1134116

**Published:** 2023-07-24

**Authors:** Jiancheng Hou, Chuansheng Chen, Qi Dong

**Affiliations:** ^1^Research Center for Cross-Straits Cultural Development, Fujian Normal University, Fuzhou, Fujian, China; ^2^State Key Lab of Cognitive Neuroscience and Learning, Beijing Normal University, Beijing, China; ^3^School of Public Health, Indiana University Bloomington, Bloomington, IN, United States; ^4^Department of Psychological Science, University of California, Irvine, CA, United States

**Keywords:** early musical training, Gestalt, visual closure, motor-free visual perception test, non-musical cognitive ability

## Abstract

Musical training has been evidenced to facilitate music perception, which refers to the consistencies, boundaries, and segmentations in pieces of music that are associated with the Gestalt principles. The current study aims to test whether musical training is beneficial to non-musical cognitive ability with Gestalt principles. Three groups of Chinese participants (with early, late, and no musical training) were compared in terms of their performances on the Motor-Free Visual Perception Test (MVPT). The results show that the participants with early musical training had significantly better performance in the Gestalt-like Visual Closure subtest than those with late and no musical training, but no significances in other Gestalt-unlike subtests was identified (Visual Memory, Visual Discrimination, Spatial Relationship, Figure Ground in MVPT). This study suggests the benefit of early musical training on non-musical cognitive ability with Gestalt principles.

## Introduction

The Gestalt theory in psychology was developed in Germany as a reaction to Elementalism and Behaviorism (Schultz and Schultz, 2004; Kaya and Akdemir, 2016). It proposes that the operational principles of the mind and brain are parallel, holistic, and analogical, with self-organizing tendencies (Garner, 1978; Delige, 1987; Narmour, 1989; Kimchi, 1994, 2003; Darwin, 1997, 2008; van der Helm, 2004; Wagemans et al., 2012). The Gestalt theory was originally defined as the ability to form human senses, especially in terms of the visual perception and recognition of whole figures rather than a collection of simple curves or lines (Garner, 1978; Pomerantz, 1981; Rock, 1986; Kimchi, 1992, 1994; Wagemans, 1995, 1997; Wagemans et al., 2012).

Like the visual domain, music has been greatly examined through the Gestalt theory, which refers to similarity, closure, continuity, proximity, conformity, etc. (Delige, 1987; Narmour, 1989; Darwin, 1997, 2008). Pitch, rhythm, melody, and emotion, which are the major components of music organization, reveal an integration of cognitive phenomena (Lerdahl and Jackendoff, 1983; Darwin, 1997, 2008; Jackendoff and Lerdahl, 2006; Sussman, 2007). For example, the auditory features are extracted when listening to music; they then enter the auditory sensory system (or echoic memory) and form a representation of auditory Gestalt (Griffiths and Warren, 2004; Koelsch, 2011). Tonal Pitch Space, which was put forward by Lerdahl (2001) and emphasizes the understanding to music perception, is primarily based on the Gestalt principles of similarity and proximity (Klumpenhouwer, 2002).

The benefits of musical training to musical abilities (e.g., pitch, rhythm, melody perceptions) and some non-musical abilities (e.g., language, math, spatial reasoning) have been widely confirmed. Based on the Gestalt and musicology theories, individuals with musical training experience are able to accurately employ the concept of fugue (a polyphonic music genre with independent lines that can simultaneously unfold in different relationships; see Levi, 1978) in their music perception (Jacobs, 1960). Deliege and Ahmadi (1990) found that individuals with musical training show more consistency when they perceive a boundary or segmentation in a music auditory stream compared to untrained individuals. Deliege et al. (1996) and Mungan et al. (2017) found that musicians tend to employ declarative knowledge about tonal relations when they play a coherent piano piece. Clarke and Krumhansl (1990) found that musicians can strongly evaluate surface-related features (e.g., rhythm, contour, pauses) and boundaries when they listen to a Stockhausen piano piece.

However, as far as we know, no study has examined whether musical training experience could benefit non-musical cognitive ability linked to the Gestalt principles. Moreover, previous studies have evidenced the different effects of early and late musical training because early training may have greater effects on brain function or structure than late training (Hensch, 2005; Steele et al., 2013), such as improved maturation in the sensorimotor region or enhanced white matter in the corpus callosum (Steele et al., 2013), which may facilitate non-musical cognitive abilities (e.g., language, attention, decision-making; Steele et al., 2013; Miendlarzewska and Trost, 2014; Hou et al., 2017). Therefore, the current study aims to test the hypothesis that early musical training is beneficial to non-musical cognitive ability linked to the Gestalt principles compared to later and no musical training. Visual Closure, one subtest from the Motor-Free Visual Perception Test (MVPT), was used as a test because it refers to the Gestalt-like ability to perceive a whole figure when fragments are missing (McCane-Bowling, 2006).

## Methods

### Participants

Data for this study came from a larger project (see He et al., 2010; Chen et al., 2013; Hou et al., 2014, 2015). A total of 563 undergraduates at Beijing Normal University participated in the MVPT and other cognitive tests (see below for details). Among them, 42 participants (11 men and 31 women) had early musical training (e.g., keyboard, piano, violin, and accordion) starting before the age of 7 years (see Table 1); 49 participants (11 men and 38 women) had late musical training (e.g., piano, clarinet, keyboard, and saxophone) starting after the age of 8 years (see Table 1). From 472 participants who had no musical training, 60 participants (11 men and 49 women; see Table 1) were selected to roughly match the early and late training participants in terms of age, gender, and IQ.

**Table 1:** Characteristics and test performance of the participants.

Characteristics	Index	Musical training group		*F_(2,148)_*		*p*	*η* ^2^	*MSE*
		Early	Late	None				
Age in years	Years	20.45 (1.10)^a^	20.14 (1.26)	20.55 (1.31)	0.86	0.31	0.01	5.90
Gender (men/women)^b^	Number	42 (11/31)	49 (11/38)	60 (11/49)				
Handedness		All right-handed	All right-handed	All right-handed				
Age at start of musical training in years	Year	5.52 (0.63)	10.66 (1.71)		64.32	0.00	0.44	549.39
Years of training	Years	5.33 (3.82)	2.82 (2.34)		13.46	0.00	0.13	130.87
*Test performances*								
IQ	IQ score	128.48 (5.97)	128.19 (7.55)	127.05 (8.25)	1.04	0.33	0.02	56.26
Visual closure	Accuracy rate	95.05 (6.32)	90.42 (8.82)	90.58 (11.26)	3.62	0.03	0.05	0.01
Visual memory	Accuracy rate	85.15 (11.53)	84.26 (10.08)	84.91 (11.23)	0.08	0.92	0.01	0.01
Visual discrimination	Accuracy rate	93.07 (8.47)	90.17 (12.53)	88.40 (11.90)	2.11	0.13	0.03	0.01
Spatial relationship	Accuracy rate	84.76 (19.66)	83.27 (21.35)	81.72 (23.48)	0.24	0.79	0.00	0.05
Figure ground	Accuracy rate	54.76 (26.16)	57.14 (23.09)	51.38 (22.20)	0.80	0.45	0.01	0.06

a Shown in parentheses are SD (except for gender distribution).

b Gender distribution did not differ by group, *χ*^2^ = 3.22, *p* = 0.23.

### Measures

Musical training history: Participants were asked about the age at which they started formal musical training, the types of musical instruments they used, and the number of years for which they had undergone formal training (see Hou et al., 2017).

MVPT: The MVPT (version 3) is used to test the ability of visual perception without motor involvement. Five categories of visual perception were tested: Visual Closure, Visual Memory, Visual Discrimination, Spatial Relationship, and Figure Ground. Visual Closure involves the Gestalt-like ability to perceive a whole figure when fragments are missing; Visual Memory involves the ability to recognize a stimulus following a brief interval; Visual Discrimination requires the ability to discriminate between salient object features; Spatial Relationship involves the accurate perception of one object amongst other objects; Figure Ground refers to the ability to discriminate an object from its background (Brown et al., 2003; McCane-Bowling, 2006). Cronbach’s alpha is 0.90 or above (Colarusso and Hammill, 2003; Zhu et al., 2010). A line drawing was shown to the participants and they were asked to choose the matching one from a set of four presented drawings. The accuracy rate for each item was used as the statistical index (see Hou et al., 2022).

Wechsler Adult Intelligence Scale: The Wechsler Adult Intelligence Scale (WAIS) was used to test intelligence. From the total 11 subtests in the original WAIS, 3 verbal subtests (General Information, Similarity, and Digit Span) and 3 performance subtests (Block Design, Picture Completion, and Digit Symbols) were adopted in the WAIS-III Chinese Version that we used here. During testing, Digit Span, General Information, Picture Completion, and Similarity were verbally administered, Digit Symbols were measured with paper and pencil, whereas the Block Design was administered with blocks. The WAIS was individually tested, and the raw scores of each participant were transformed into a standardized IQ score (see Hou et al., 2014, 2017, 2022).

## Results

From Table 1, the three subgroups of participants were successfully matched because they had no differences in age, gender, and IQ. Moreover, the early training group had significantly more training years than the late training group.

**Figure 1:**
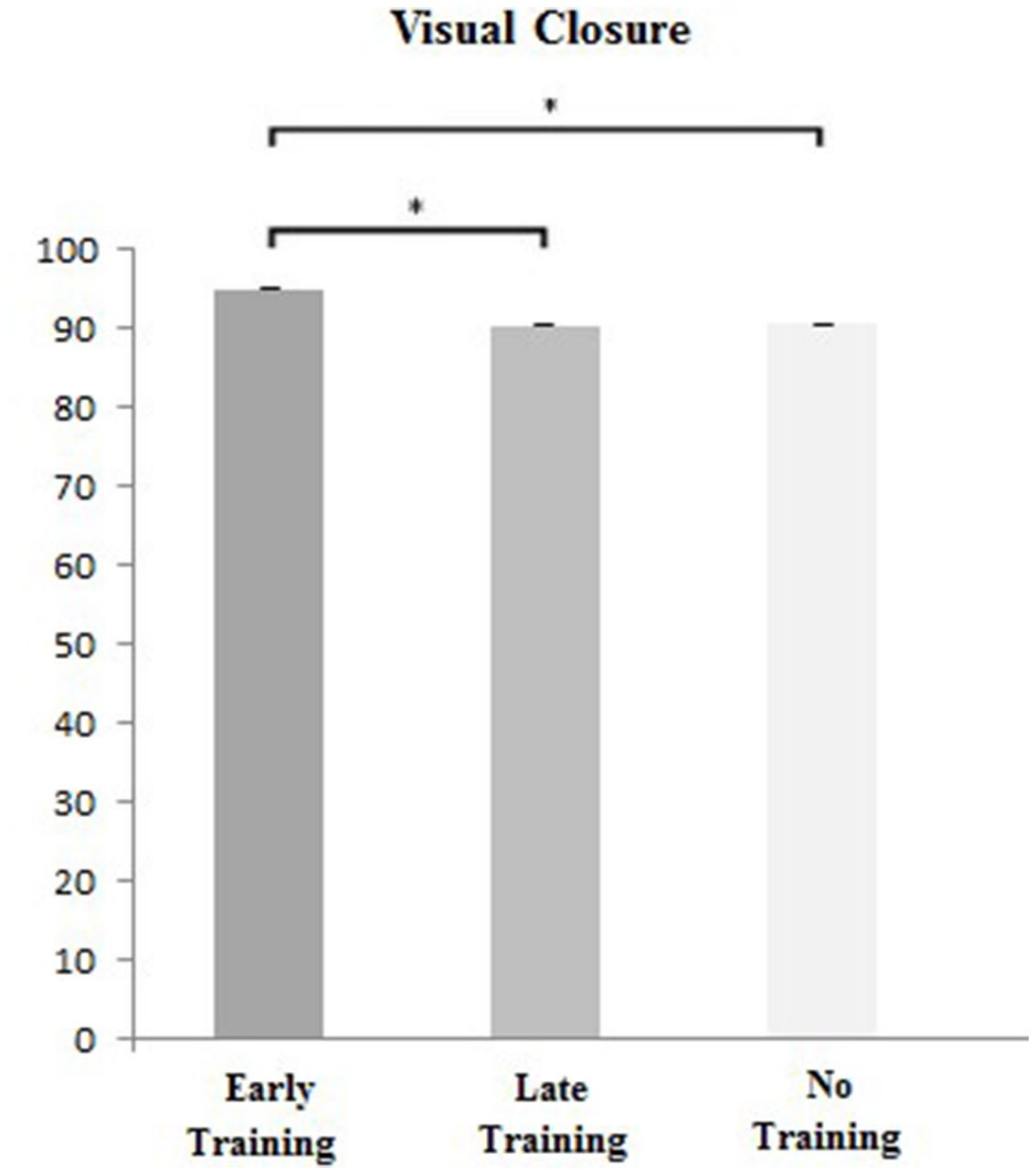
Visual closure scores of the three groups. Error bars indicate standard errors. *p < 0.05.

One-way ANOVA analysis showed that in each item of MVPT, the three subgroups only significantly differed in Visual Closure (see Table 1). *Post hoc* analysis showed that the participants with early musical training performed significantly better in Visual Closure than those with late and no musical training (early vs. late: *p* = 0.02; early vs. no: p = 0.02). Participants with late and no musical training showed no significant differences in their Visual Closure performance (*p* = 0.93) (see Figure 1). Moreover, the three subgroups had no significances in the other four MVPT subitems (see Table 1).

Table 2 shows the intercorrelations among the variables. Age at start of training was significantly correlated with years of training and Visual Discrimination. Years of training was significantly correlated with Visual Closure. Visual Closure was significantly correlated with Visual Memory, Visual Discrimination, Spatial Relationship, Figure Ground, and IQ. There were also significant correlations between related constructs, specifically, between Visual Memory and Visual Discrimination/Figure Ground/IQ, respectively; between Visual Discrimination and Spatial Relationship/Figure Ground/IQ, respectively; between Spatial Relationship and Figure Ground; and between Figure Ground and IQ.

**Table 2:** Intercorrelations (Pearson’s *r*) among major variables.

Variables	Years of training	IQ	MVPT				
			Visual closure	Visual memory	Visual discrimination	Spatial relationship	Figure ground
Age at start of training	−0.64^**^	0.02	−0.121	−0.04	−0.18^*^	−0.07	−0.12
Years of training		−0.10	0.19^*^	0.03	0.12	0.00	−0.06
IQ			0.19^*^	0.25^**^	0.20^*^	0.15	0.18^*^
Visual closure				0.20^*^	0.45^**^	0.24^**^	0.27^**^
Visual memory					0.18^*^	0.14	0.20^*^
Visual discrimination						0.40^**^	0.33^**^
Spatial relationship	-	-					0.33^**^

*N* = 151. For the purpose of correlational analyses, years of training was assigned 0 for the participants who had no musical training, and, correspondingly, age at start of training was assigned the average age of 20 years. ^*^ *p* < 0.05, ^**^ *p* < 0.01.

Because years of training, IQ, and four other MVPT items were significantly correlated with Visual Closure, analysis of covariance was performed to examine whether group differences remained after controlling for years of training, IQ, and the other four items. The results showed that the inclusion of these variates affected the group differences in Visual Closure (*F*_(2,148)_ = 2.12, *p* = 0.12, *η^2^* = 0.03, *MSE* = 0.01).

## Discussion

Many studies have confirmed the effect of musical training (George and Coch, 2011; Wong and Gauthier, 2012; Matthews et al., 2018), even late or short musical training (Wong et al., 2019; Che et al., 2022; Zanto et al., 2022), on visual perception, but no study has examined the effect of musical training on visual perception associated with the Gestalt principles as far as we know. Through the Visual Closure subset from the MVPT, the current study confirms our hypothesis that, unlike late musical training, early musical training is beneficial to non-musical cognitive ability associated with Gestalt-like ability.

Unlike other visual perceptual skills, such as Visual Discrimination (the ability to recognize similarities and differences between shapes, sizes, colors, objects, and patterns), Figure Ground (the ability to filter visual information that is not important so that an individual can focus on the relevant visual information), and Visual Memory (the ability to immediately recall what the eye has seen), Visual Closure allows an individual to know what an object is even when the object is only partially visible, and it is necessary to quickly view objects and mentally determine what they are before an individual sees the entire object (Colarusso and Hammill, 2003; McCane-Bowling, 2006). This is similar to a separation or boundary in music, which is usually located at a place that plays a crucial role in a piece of music and can catch the listener’s attention, allowing them to extract information for a better understanding of the piece (Mungan et al., 2017). The attention of the listener is caught at a more implicit level in non-musicians but at a relatively explicit level in musicians (Bigand and Poulin-Charronnat, 2006; Mungan et al., 2017). Indeed, early musical training can improve the ability of attention processing (Schlaug et al., 2005; Moreno et al., 2009; Posner and Patoine, 2009; Kraus et al., 2012, 2014; Nutley et al., 2013). This might explain why early musical training could drive better performance on music segmentations with a more top-down and explicit method (Mungan et al., 2017) that could possibly transfer to non-musical cognitive performance.

Moreover, the Gamma band (γ), a physiological index through the electroencephalography (EEG) measure, reflects numerous neural oscillations and synchronization in the high-frequency range (>30 Hz) an enhanced ability of cognitive integration, and it can also detect phase synchronization (or synchrony) (Singer and Gray, 1995; Rodriguez et al., 1999; Mormann et al., 2000; Bhattacharya et al., 2001; Jausovec and Habe, 2003; Hou and Liu, 2009; Urakami et al., 2013; Tseng et al., 2019). Its induced evocation or oscillations not only represent the attention and memory representations or other cognitive functions (Herrmann, 2001; Bauer et al., 2006; Poikonen, 2018), but also be responsible for Gestalt processing (Keil et al., 1999; Herrmann, 2001; Kaiser and Lutzenberger, 2003; Griffiths and Warren, 2004; Lachaux et al., 2005; Sedley and Cunningham, 2013; Sanyal et al., 2017; Poikonen, 2018). Some studies have shown that, compared to non-musicians, early musical training in musicians induces Gamma significance during music listening (Bhattacharya et al., 2001; Bhattacharya and Petsche, 2001) or music imagery (Urakami et al., 2013) or when they perform a classical Shepard-Metzler mental rotation task (Bhattacharya et al., 2001). This is possibly because musical training may integrate implicit music memory (Bhattacharya et al., 2001; Bhattacharya and Petsche, 2001; Urakami et al., 2013) or direct internal self-reference (Urakami et al., 2013) or bind together several features of the intrinsic complexity of music in a dynamic way (Bhattacharya et al., 2001).

Furthermore, early musical training has preferable impacts on brain function and structure compared to late musical training (Hensch, 2005; Steele et al., 2013). A brain white matter study found that early musical training (before 7 years old) significantly facilitates neural maturity, such as the sensorimotor cortex (Steele et al., 2013) that is associated with the processing of attention and top-down control (Sumner et al., 2006; Witt and Stevens, 2013; Belkaid et al., 2017; Morillon and Baillet, 2017), both of which are linked to Gestalt-like ability (Li and Logan, 2008; Shen et al., 2013; Zaretskaya et al., 2013; Katsuki and Constantinidis, 2014; Marini and Marzi, 2016). Moreover, early musical training can biochemically improve the level of dopamine D4 receptors in the prefrontal region of the brain (Nemirovsky et al., 2009; Cocker et al., 2014; Miendlarzewska and Trost, 2014), and dopamine expression can enhance executive control or attention (Durston et al., 2005; Bakermans-Kranenburg et al., 2008; Barnes et al., 2011; Sweitzer et al., 2013; Cleveland et al., 2015; Qian et al., 2018).

We also found that, although age and IQ in the three groups were successfully matched, the effect of early musical training on Visual Closure was not independent of years of musical training, IQ, and four other subitems; after controlling for these variates, the effect of early training on Visual Closure performance did not significantly remain in this study. McCane-Bowling (2006) showed that overall visual perceptual ability relies on the five subtests that have interrelated processes in MVPT. Indeed, our study also shows their significant intercorrelations. However, all the MVPT subitems, except Visual Closure, showed no significance among the three subgroups in our study. Moreover, the start time of training (early vs. late) seems to be insignificant. This is consistent with some previous studies that showed that age at the start of musical training (before vs. after the age of 7) is not associated with other cognitive abilities, such as vocabulary, IQ, reasoning, digit span, and letter-number sequences (Bailey and Penhune, 2010, 2012; Hou et al., 2017). Therefore, the deeper inner relationship between IQ, duration of musical training, some kinds of visual perception, and Gestalt-like visual processing should be further investigated.

The current study has some limitations. First, the three groups differed not only in early vs. late vs. no musical training but also in terms of musical instruments, music style, and training frequency. Therefore, these potential variables may covary with early vs. late training; for example, early training may be of higher frequency than late training, or early training may generally refer to the piano, whereas late training may refer to wind instruments (Hou et al., 2017). Second, participants in the current study were Chinese non-musicians, i.e., they were not music majors in college, were not making a living as a musician, and did not appear to become professional musicians; thus, this study should be extended to professional musicians with other cultural backgrounds, and further examination is needed.

In summary, early musical training is associated with better non-musical cognitive ability with Gestalt principles. Replicating the current study in populations with other cultural backgrounds and examining the related neural basis are needed.

## Data availability statement

The raw data supporting the conclusions of this article will be made available by the authors, without undue reservation.

## Ethics statement

The studies involving human participants were reviewed and approved by the Institutional Review Board of the State Key Lab of Cognitive Neuroscience and Learning at Beijing Normal University. The patients/participants provided their written informed consent to participate in this study.

## Author contributions

JH put forward the research idea, wrote the manuscript, organized the manuscript structure, and revised the manuscript. CC designed and organized the experiments, revised the manuscript, and applied for funding for the project. QD designed and organized the experiments, revised the manuscript, and applied for the funding for the project. All authors contributed to the article and approved the submitted version.

## Funding

This study was supported by the 111 Project from the Ministry of Education of China (B07008).

## Conflict of interest

The authors declare that the research was conducted in the absence of any commercial or financial relationships that could be construed as a potential conflict of interest.

## Publisher’s note

All claims expressed in this article are solely those of the authors and do not necessarily represent those of their affiliated organizations, or those of the publisher, the editors and the reviewers. Any product that may be evaluated in this article, or claim that may be made by its manufacturer, is not guaranteed or endorsed by the publisher.

## References

[ref1] Bailey J. A. Penhune V. B. (2010). Rhythm synchronization performance and auditory working memory in early- and late-trained musicians. Exp. Brain Res. 204, 91–101. doi: 10.1007/s00221-010-2299-y, PMID: 20508918

[ref01] Bailey J. Penhune V. B. (2012). A sensitive period for musical training: contributions of age of onset and cognitive abilities. Anna. N. Y. Acad. Sci. 1252, 163–170. doi: 10.1111/j.1749-6632.2011.06434.x 22524355

[ref2] Bakermans-Kranenburg M. J. Van IJzendoorn M. H. Pijlman F. T. Mesman J. Juffer F. (2008). Experimental evidence for differential susceptibility: dopamine D4 receptor polymorphism (DRD4 VNTR) moderates intervention effects on toddlers’ externalizing behavior in a randomized controlled trial. Dev. Psychol. 44, 293–300. doi: 10.1037/0012-1649.44.1.293, PMID: 18194028

[ref3] Barnes J. J. Dean A. J. Nandam L. S. O’Connell R. G. Bellgrove M. A. (2011). The molecular genetics of executive function: role of monoamine system genes. Biol. Psychiatry 69, e127–e143. doi: 10.1016/j.biopsych.2010.12.040, PMID: 21397212

[ref4] Bauer M. Oostenveld R. Peeters M. Fries P. (2006). Tactile spatial attention enhances gamma-band activity in somatosensory cortex and reduces low-frequency activity in parieto-occipital areas. J. Neurosci. 26, 490–501. doi: 10.1523/JNEUROSCI.5228-04.2006, PMID: 16407546 PMC6674422

[ref5] Belkaid M. Cuperlier N. Gaussier P. (2017). Emotional metacontrol of attention: top-down modulation of sensorimotor processes in a robotic visual search task. PLoS One 12:e0184960. doi: 10.1371/journal.pone.0184960, PMID: 28934291 PMC5608313

[ref6] Bhattacharya J. Petsche H. (2001). Enhanced phase synchrony in the electroencephalograph gamma band for musicians while listening to music. Phys. Rev. E 64:012902. doi: 10.1103/PhysRevE.64.012902, PMID: 11461312

[ref7] Bhattacharya J. Petsche P. Feldmann U. Rescher B. (2001). EEG gamma-band phase synchronization between posterior and frontal cortex during mental rotation in humans. Neurosci. Lett. 311, 29–32. doi: 10.1016/S0304-3940(01)02133-4, PMID: 11585560

[ref8] Bigand E. Poulin-Charronnat B. (2006). Are we “experienced listeners”? A review of the musical capacities that do not depend on formal musical training. Cognition 100, 100–130. doi: 10.1016/j.cognition.2005.11.007, PMID: 16412412

[ref10] Brown G. T. Rodger S. Davis A. (2003). Motor-free visual perception test—revised: an overview and critique. Br. J. Occup. Ther. 66, 159–167. doi: 10.1177/030802260306600405

[ref13] Che Y. Jicol C. Ashwin C. Petrini K. (2022). An RCT study showing few weeks of music lessons enhance audio-visual temporal processing. Sci. Rep. 12:20087. doi: 10.1038/s41598-022-23340-4, PMID: 36418441 PMC9684138

[ref14] Chen C. S. Chen C. H. Moyzis R. He Q. Lei X. Li J.. (2013). Genotypes over-represented among college students are linked to better cognitive abilities and socioemotional adjustment. Cult. Brain 1, 47–63. doi: 10.1007/s40167-013-0003-3

[ref15] Clarke E. F. Krumhansl C. L. (1990). Perceiving musical time. Music. Percept. 7, 213–251. doi: 10.2307/40285462

[ref16] Cleveland H. H. Schlomer G. L. Vandenbergh D. J. Feinberg M. Greenberg M. Spoth R.. (2015). The conditioning of intervention effects on early adolescent alcohol use by maternal involvement and dopamine receptor D4 (DRD4) and serotonin transporter linked polymorphic region (5-HTTLPR) genetic variants. Dev. Psychopathol. 27, 51–67. doi: 10.1017/S0954579414001291, PMID: 25640830 PMC4450765

[ref17] Cocker P. J. Le Foll B. Rogers R. D. Winstanley C. A. (2014). A selective role for dopamine D4 receptors in modulating reward expectancy in a rodent slot machine task. Biol. Psychiatry 75, 817–824. doi: 10.1016/j.biopsych.2013.08.026, PMID: 24094512

[ref18] Colarusso R. P. Hammill D. D. (2003). Motor-free visual perception test. (3rd). Novata, CA: Academic Therapy Publications

[ref19] Darwin C. J. (1997). Auditory grouping. Trends Cogn. Sci. 1, 327–333. doi: 10.1016/S1364-6613(97)01097-8 21223942

[ref20] Darwin C. J. (2008). Listening to speech in the presence of other sounds. Philos. Trans. R. Soc. B 363, 1011–1021. doi: 10.1098/rstb.2007.2156, PMID: 17827106 PMC2606793

[ref21] Deliege I. Ahmadi A. (1990). Mechanisms of cue extraction in musical groupings: a study of perception on Sequenza VI for viola solo by Luciano Berio. Psychol. Music 18, 18–44. doi: 10.1177/0305735690181003

[ref22] Deliege I. Melen M. Stammers D. Cross I. (1996). Musical schemata in real-time listening to a piece of music. Music. Percept. 14, 117–159. doi: 10.2307/40285715

[ref23] Delige I. (1987). Grouping conditions in listening to music: an approach to Lerdahl and Jackendoff’s grouping preference rules. Music. Percept. 4, 325–359. doi: 10.2307/40285378

[ref24] Durston S. Fossella J. A. Casey B. J. Hulshoff H. E. Galvan A. Schnack H. G. van Engeland H. (2005). Differential effects of DRD4 and DAT1 genotype on fronto-striatal gray matter volumes in a sample of subjects with attention deficit hyperactivity disorder, their unaffected siblings, and controls. Mol. Psychiatry, 10, 678–685. doi: 10.1038/sj.mp.4001649, PMID: 15724142

[ref26] Garner W. R. (1978). “Aspects of a stimulus: features, dimensions, and configurations” in Cognition and categorization. eds. Rosch E. Lloyd B. B. (Hillsdale, NJ: Erlbaum), 99–133.

[ref27] George E. M. Coch D. (2011). Music training and working memory: an ERP study. Neuropsychologia 49, 1083–1094. doi: 10.1016/j.neuropsychologia.2011.02.001, PMID: 21315092

[ref28] Griffiths T. D. Warren J. D. (2004). What is an auditory object? Nat. Rev. Neurosci. 5, 887–892. doi: 10.1002/1527-2648(20020806)4:83.0.CO;2-R 15496866

[ref29] He Q. Xue G. Chen C. Lu Z. Dong Q. Lei X.. (2010). Serotonin transporter gene-linked polymorphic region (5-HTTLPR) influences decision making under ambiguity and risk in a large Chinese sample. Neuropharmacology 59, 518–526. doi: 10.1016/j.neuropharm.2010.07.008, PMID: 20659488 PMC2946467

[ref30] Hensch T. K. (2005). Critical period plasticity in local cortical circuits. Nat. Rev. Neurosci. 6, 877–888. doi: 10.1038/nrn1787 16261181

[ref31] Herrmann C. S. (2001). Human EEG responses to 1-100 Hz flicker: resonance phenomena in visual cortex and their potential correlation to cognitive phenomena. Exp. Brain Res. 137, 346–353. doi: 10.1007/s002210100682, PMID: 11355381

[ref33] Hou J. Chen C. Dong Q. (2015). Resting-state functional connectivity and pitch identification ability in non-musicians. Front. Neurosci. 9:7. doi: 10.3389/fnins.2015.00007, PMID: 25717289 PMC4324073

[ref34] Hou J. Chen C. Dong Q. Prabhakaran V. Nair V. (2022). Superior pitch identification ability is associated with better mental rotation performance. Musicae Scientiae 27, 117–136. doi: 10.1177/10298649211013409

[ref35] Hou J. Chen C. Wang Y. Liu Y. He Q. Li J.. (2014). Superior pitch identification ability is associated with better executive functions. Psychomusicology 24, 136–146. doi: 10.1037/a0036963

[ref36] Hou J. He Q. Chen C. Dong Q. (2017). Early musical training contributes to decision-making ability. Psychomusicology 27, 75–80. doi: 10.1037/pmu0000174

[ref37] Hou J. Liu C. (2009). The review about brain’s physiological action induced by different music activities. J. Central Conserv. Music 3, 137–144. (in Chinese)

[ref38] Jackendoff R. Lerdahl F. (2006). The capacity for music: what is it, and what’s special about it? Cognition 100, 33–72. doi: 10.1016/j.cognition.2005.11.005, PMID: 16384553

[ref39] Jacobs C. (1960). Psychology of music: some European studies. Acta Psychological 17, 273–297. doi: 10.1016/0001-6918(60)90022-6

[ref40] Jausovec N. Habe K. (2003). The “Mozart effect”: an electroencephalographic analysis employing the methods of induced event-related desynchronization/synchronization and event-related coherence. Brain Topogr. 16, 73–84. doi: 10.1023/B:BRAT.0000006331.10425.4b 14977200

[ref41] Kaiser J. Lutzenberger W. (2003). Induced gamma-band activity and human brain function. Neuroscientist 9, 475–484. doi: 10.1177/1073858403259137 14678580

[ref42] Katsuki F. Constantinidis C. (2014). Bottom-up and top-down attention: different processes and overlapping neural systems. Neuroscientist 20, 509–521. doi: 10.1177/1073858413514136 24362813

[ref43] Kaya Z. Akdemir S. (2016). Learning and teaching: theories, approaches and models. 2nd Edn. Ankara: Cozum Publishing.

[ref44] Keil A. Muller M. M. Ray W. J. Gruber T. Elbert T. (1999). Human gamma band activity and perception of a gestalt. J. Neurosci. 19, 7152–7161. doi: 10.1027//0269-8803.15.1.48a, PMID: 10436068 PMC6782859

[ref45] Kimchi R. (1992). Primacy of wholistic processing and global/local paradigm: a critical review. Psychol. Bull. 112, 24–38. doi: 10.1037//0033-2909.112.1.24, PMID: 1529037

[ref46] Kimchi R. (1994). The role of wholistic/configural properties versus global properties in visual form perception. Perception 23, 489–504. doi: 10.1068/p230489, PMID: 7800465

[ref47] Kimchi R. (2003). “Relative dominance of holistic and component properties in the perceptual organization of visual objects” in Perception of faces, objects, and scenes: Analytic and holistic processes. eds. Peterson M. A. Rhodes G. (New York, NY: Oxford University Press), 235–263.

[ref48] Klumpenhouwer H. (2002). “Dualist tonal space and transformation in nineteenth-century musical thought” in The Cambridge history of Western music theory. ed. Christensen T. (London: Cambridge University Press), 456–476.

[ref49] Koelsch S. (2011). Toward a neural basis of music perception: a review and updated model. Front. Psychol. 2:110. doi: 10.3389/fpsyg.2011.00110, PMID: 21713060 PMC3114071

[ref50] Kraus N. Slater J. Thompson E. C. Hornickel J. Strait D. L. Nicol T. White-Schwoch T. (2014). Music enrichment programs improve the neural encoding of speech in at-risk children. J. Neurosci., 34, 11913–11918. doi: 10.1523/JNEUROSCI.1881-14.2014, PMID: 25186739 PMC6608462

[ref51] Kraus N. Strait D. Parbery-Clark A. (2012). Cognitive factors shape brain networks for auditory skills: spotlight on auditory working memory. Ann. N. Y. Acad. Sci. 1252, 100–107. doi: 10.1111/j.1749-6632.2012.06463.x, PMID: 22524346 PMC3338202

[ref52] Lachaux J. P. George N. Tallon-Baudry C. Martinerie J. Hugueville L. Minotti L. (2005). The many faces of the gamma band response to complex visual stimuli. NeuroImage 25, 491–501. doi: 10.1016/j.neuroimage.2004.11.052, PMID: 15784428

[ref53] Lerdahl F. (2001). Tonal pitch space. Oxford: Oxford University Press.

[ref54] Lerdahl F. Jackendoff R. (1983). A generative theory of tonal music. Cambridge, MA: MIT Press.

[ref55] Levi D. S. (1978). Expressive qualities in music perception and music education. J. Res. Music. Educ. 26, 425–435. doi: 10.2307/3690713

[ref57] Li X. Logan G. D. (2008). Object-based attention in Chinese readers of Chinese words: beyond gestalt principles. Psychon. Bull. Rev. 15, 945–949. doi: 10.3758/PBR.15.5.945, PMID: 18926986

[ref58] Marini F. Marzi C. A. (2016). Gestalt perceptual organization of visual stimuli captures attention automatically: electrophysiological evidence. Front. Hum. Neurosci. 10:446. doi: 10.3389/fnhum.2016.00446, PMID: 27630555 PMC5005981

[ref59] Matthews N. Welch L. Festa E. (2018). Superior visual timing sensitivity in auditory but not visual world class drum corps experts. eNeuro 5, 1–21. doi: 10.1523/ENEURO.0241-18.2018, PMID: 30627642 PMC6325546

[ref60] McCane-Bowling S. J. (2006). Test review: motor-free visual perception test. J. Psychoeduc. Assess. 24, 265–272. doi: 10.1177/0734282906286339

[ref61] Miendlarzewska E. A. Trost W. J. (2014). How musical training affects cognitive development: rhythm, reward and other modulating variables. Front. Neurosci. 7:279. doi: 10.3389/fnins.2013.00279, PMID: 24672420 PMC3957486

[ref62] Moreno S. Marques C. Santos A. Santos M. Castro S. Besson M. (2009). Musical training influences linguistic abilities in 8-year-old children: more evidence for brain plasticity. Cereb. Cortex 19, 712–723. doi: 10.1093/cercor/bhn120, PMID: 18832336

[ref63] Morillon B. Baillet S. (2017). Motor origin of temporal predictions in auditory attention. Proc. Natl. Acad. Sci. 114, E8913–E8921. doi: 10.1073/pnas.1705373114, PMID: 28973923 PMC5651745

[ref64] Mormann F. Lehnertz K. David P. Elger C. E. (2000). Mean phase coherence as a measure for phase synchronization and its application to the EEG of epilepsy patients. Physica D 144, 358–369. doi: 10.1016/S0167-2789(00)00087-7

[ref65] Mungan E. Yazici Z. Kaya M. (2017). Perceiving boundaries in unfamiliar Turkish Makam music evidence for gestalt universals? Music. Percept. 34, 267–290. doi: 10.1525/MP.2017.34.3.267

[ref68] Narmour E. (1989). The genetic code of melody: cognitive structures generated by the implication-realization model. Contemp. Music. Rev. 4, 45–63. doi: 10.1080/07494468900640201

[ref69] Nemirovsky S. I. Avale M. E. Brunner D. Rubinstein M. (2009). Reward-seeking and discrimination deficits displayed by hypodopaminergic mice are prevented in mice lacking dopamine D4 receptors. Synapse 63, 991–997. doi: 10.1002/syn.20680, PMID: 19598175

[ref70] Nutley S. B. Darki F. Klingberg T. (2013). Music practice is associated with development of working memory during childhood and adolescence. Front. Hum. Neurosci. 7:926. doi: 10.3389/fnhum.2013.00926, PMID: 24431997 PMC3882720

[ref71] Poikonen H. (2018). Dance on cortex erps and phase synchrony in dancers and musicians during a contemporary dance piece. Doctoral dissertation, Helsinki: University of Helsinki.

[ref72] Pomerantz J. R. (1981). “Perceptual organization in information processing” in Perceptual Organization. eds. Pomerantz J. R. Kubovy M. (Hillsdale, NJ: Erlbaum), 141–180.

[ref73] Posner M. I. Patoine B. (2009). How arts training improves attention and cognition. Cerebrum Available at: http://dana.org/news/cerebrum/detail.aspx?id=23206

[ref75] Qian A. Wang X. Liu H. Tao J. Zhou J. Ye Q.. (2018). Dopamine D4 receptor gene associated with the frontal-striatal-cerebellar loop in children with ADHD: a resting-state fMRI study. Neurosci. Bull. 34, 497–506. doi: 10.1007/s12264-018-0217-7, PMID: 29564731 PMC5960453

[ref76] Rock I. (1986). “The description and analysis of object and event perception” in Handbook of perception and human performance, 33. eds. Boff K. R. Kaufman L. Thomas J. P. (New York, NY: Wiley), 1–71.

[ref77] Rodriguez E. George N. Lachaux J. P. Martinerie J. Renault B. Varela F. J. (1999). Perception’s shadow: long distance synchronization of human brain activity. Nature 397, 430–433. doi: 10.1038/17120, PMID: 9989408

[ref78] Sanyal S. Banerjee A. Roy S. Sengupta S. Biswas S. Nag S.. (2017). Gestalt phenomenon in music? A neurocognitive physics study with EEG. Computer Science, Available at: https://arxiv.org/abs/1703.06491

[ref79] Schlaug G. Norton A. Overy K. Winner E. (2005). Effects of music training on the child’s brain and cognitive development. Ann. N. Y. Acad. Sci. 1060, 219–230. doi: 10.1196/annals.1360.015, PMID: 16597769

[ref80] Schultz D. P. Schultz E. S. (2004). A history of modern psychology (8th). Belmont, CA: Wadsworth/Thomson, 363–378.

[ref81] Sedley W. Cunningham M. O. (2013). Do cortical gamma oscillations promote or suppress perception? An under-asked question with an over-assumed answer. Front. Hum. Neurosci. 7:595. doi: 10.3389/fnhum.2013.00595, PMID: 24065913 PMC3778316

[ref82] Shen J. Ojha A. Lee M. (2013). Role of gestalt principles in selecting attention areas for object recognition. International Conference on Neural Information Processing, 90–97.

[ref83] Singer W. Gray C. M. (1995). Visual feature integration and the temporal correlation hypothesis. Annu. Rev. Neurosci. 18, 555–586. doi: 10.1146/annurev.ne.18.030195.003011, PMID: 7605074

[ref84] Steele C. J. Bailey J. A. Zatorre R. J. Penhune V. B. (2013). Early musical training and white-matter plasticity in the corpus callosum: evidence for a sensitive period. J. Neurosci. 33, 1282–1290. doi: 10.1523/JNEUROSCI.3578-12.2013, PMID: 23325263 PMC6704889

[ref85] Sumner P. Tsai P. Yu K. Nachev P. (2006). Attentional modulation of sensorimotor processes in the absence of perceptual awareness. Proc. Natl. Acad. Sci. 103, 10520–10525. doi: 10.1073/pnas.0601974103, PMID: 16793924 PMC1502490

[ref86] Sussman E. (2007). A new view on the MMN and attention debate: the role of context in processing auditory events. J. Psychophysiol. 21, 164–175. doi: 10.1027/0269-8803.21.34.164

[ref87] Sweitzer M. M. Halder I. Flory J. D. Craig A. E. Gianaros P. J. Ferrell R. E.. (2013). Polymorphic variation in the dopamine D4 receptor predicts delay discounting as a function of childhood socioeconomic status: evidence for differential susceptibility. Soc. Cogn. Affect. Neurosci. 8, 499–508. doi: 10.1093/scan/nss020, PMID: 22345368 PMC3682430

[ref88] Tseng Y. L. Liu H. H. Liou M. Tsai A. C. Chien V. Shyu S.. (2019). Lingering sound: event-related phase-amplitude coupling and phase-locking in fronto-temporo-parietal functional networks during memory retrieval of music melodies. Front. Hum. Neurosci. 13:150. doi: 10.3389/fnhum.2019.00150, PMID: 31178706 PMC6538802

[ref89] Urakami Y. Kawamura K. Washizawa Y. Cichocki A. (2013). Electroencephalographic gamma-band activity and music perception in musicians and non-musicians. Act. Nerv. Super. Rediviva 55, 149–157.

[ref90] van der Helm P. A. (2004). Transparallel processing by hyperstrings. Proc. Natl. Acad. Sci. 101, 10862–10867. doi: 10.1073/pnas.0403402101, PMID: 15263075 PMC503711

[ref91] Wagemans J. (1995). Detection of visual symmetries. Spat. Vis. 9, 9–32. doi: 10.1163/156856895X00098 7626549

[ref92] Wagemans J. (1997). Characteristics and models of human symmetry detection. Trends Cogn. Sci. 1, 346–352. doi: 10.1016/S1364-6613(97)01105-4 21223945

[ref93] Wagemans J. Feldman J. Gepshtein S. Kimchi R. Pomerantz J. van der Helm P.. (2012). A century of gestalt psychology in visual perception II. Conceptual and theoretical foundations. Psychol. Bull. 138, 1218–1252. doi: 10.1037/a0029334, PMID: 22845750 PMC3728284

[ref94] Witt S. T. Stevens M. C. (2013). The role of top-down control in different phases of a sensorimotor timing task: a DCM study of adults and adolescents. Brain Imaging Behav. 7, 260–273. doi: 10.1007/s11682-013-9224-5, PMID: 23475755 PMC3743949

[ref95] Wong Y. K. Gauthier I. (2012). Music-reading expertise alters visual spatial resolution for musical notation. Psychon. Bull. Rev. 19, 594–600. doi: 10.3758/s13423-012-0242-x, PMID: 22460744 PMC3394230

[ref96] Wong A. C. N. Ng T. Y. K. Lui K. F. H. Yip K. H. M. Wong Y. K. (2019). Visual training with musical notes changes late but not early electrophysiological responses in the visual cortex. J. Vis. 19:8. doi: 10.1167/19.7.8, PMID: 31318402

[ref97] Zanto T. P. Johnson V. Ostrand A. Gazzaley A. (2022). How musical rhythm training improves short-term memory for faces. Proc. Natl. Acad. Sci. 119:e2201655119. doi: 10.1073/pnas.2201655119, PMID: 36191231 PMC9564217

[ref98] Zaretskaya N. Anstis S. Bartels A. (2013). Parietal cortex mediates conscious perception of illusory gestalt. J. Neurosci. 33, 523–531. doi: 10.1523/jneurosci.2905-12.2013, PMID: 23303932 PMC6704896

[ref99] Zhu B. Chen C. Loftus E. F. Lin C. He Q. Chen C.. (2010). Individual differences in false memory from misinformation: cognitive factors. Memory 18, 543–555. doi: 10.1080/09658211.2010.487051, PMID: 20623420

